# Lipidomics reveals altered biosynthetic pathways of glycerophospholipids and cell signaling as biomarkers of the polycystic ovary syndrome

**DOI:** 10.18632/oncotarget.23393

**Published:** 2017-12-17

**Authors:** Mariona Jové, Irene Pradas, Alba Naudí, Susana Rovira-Llopis, Celia Bañuls, Milagros Rocha, Manuel Portero-Otin, Antonio Hernández-Mijares, Victor M. Victor, Reinald Pamplona

**Affiliations:** ^1^ Department of Experimental Medicine, Lleida University-Institute for Research in Biomedicine of Lleida (UdL-IRBLleida), 25198 Lleida, Spain; ^2^ Foundation for the Promotion of Healthcare and Biomedical Research in the Valencian Community (FISABIO), Service of Endocrinology, University Hospital Dr. Peset, 46017 Valencia, Spain; ^3^ Fundación Investigación Hospital Clínico Universitario/INCLIVA, Valencia University, 46010 Valencia, Spain; ^4^ Department of Medicine, Valencia University, 46010 Valencia, Spain; ^5^ Department of Physiology, Valencia University, 46010 Valencia, Spain

**Keywords:** cell signaling molecules, glycerophospholipids, free fatty acids, lipidomics, lipid de novo biosynthesis

## Abstract

**Purpose:**

In this work, a non-targeted approach was used to unravel changes in the plasma lipidome of PCOS patients. The aim is to offer new insights in PCOS patients strictly selected in order to avoid confounding factors such as dyslipemia, obesity, altered glucose/insulin metabolism, cardiovascular disease, or cancer.

**Results:**

Multivariate statistics revealed a specific lipidomic signature for PCOS patients without associated pathologies. This signature implies changes, mainly by down-regulation, in glycerolipid, glycerophospholipid and sphingolipid metabolism suggesting an altered biosynthetic pathway of glycerophospholipids and cell signaling as second messengers in women with PCOS.

**Conclusions:**

Our study confirms that a lipidomic approach discriminates a specific phenotype from PCOS women without associated pathologies from healthy controls.

**Methods:**

In a cross-sectional pilot study, data were obtained from 34 subjects, allocated to one of two groups: a) lean, healthy controls (*n* = 20), b) PCOS patients (*n* = 14) with diagnosis based on hyperandrogenaemia, oligo-anovulation and abnormal ovaries with small follicular cysts. A detailed biochemical characterization was made and lipidomic profiling was performed via an untargeted approach using LC-ESI-QTOF MS/MS.

## INTRODUCTION

Polycystic ovary syndrome (PCOS) is an endocrine-metabolic disorder in women of reproductive age with a prevalence, in dependence on the study population, of 5–20% [1–11]. The key basic features for its diagnosis are hyperandrogenism, chronic anovulation, and polycystic ovaries [12–18]. PCOS pathology is often associated with obesity [19–24], insulin resistance [21, 23, 25, 26], type II diabetes [11, 27], cardiovascular disease, metabolic syndrome [28–34], and gynecological cancer [35–38]. Female infertility and pregnancy loss are additional consequences of this disorder. In addition, PCOS has been also related to an increased oxidative stress and leukocyte-endothelium interactions, suggesting mitochondrial dysfunction and cardiovascular events under this condition [39, 40].

Although hyperandrogenism is one of the underlying key factors in this syndrome [15, 41, 42], likely as the result of a primary defect in steroidogenesis [21], the pathological conditions associated with PCOS complicate the understanding of the disease's origin becoming a big challenge for both clinical and scientific communities.

The recent development of comprehensive omics approaches has provided an opportunity to address open questions about this disorder. Thus, genomics, transcriptomics, and proteomics approaches suggest the existence of altered pathways that affect protein folding, cytoskeleton, immune response, inflammation, iron metabolism, fibrinolysis and thrombosis, TGF-beta pathway, insulin signaling pathway, intracellular calcium metabolism, and oxidative stress, among others, which could play a role in the pathophysiology of PCOS [43, 44]. At present moment, however, no conclusive results have been obtained and thus, no mechanistic conclusions can be formulated.

More recently, metabolomics has offered a new perspective to study PCOS [44, 45], because it is closer to the actual phenotype than either both genomics/transcriptomics or proteomics. This approach allows to identify in plasma/serum and urine samples metabolites involved mostly in carbohydrate, lipid, and amino acid metabolism, as well as steroid hormone metabolism, as potential biomarkers for different PCOS phenotypes [46–49] or PCOS patients with other pathologies associated (e.g. overweight/obesity [48, 50] or insulin resistance or type 2 diabetes [51–55]). Interestingly, a recent and unique metabolomic study centered in follicular niche demonstrated that mitochondrial dysfunction of cumulus cells can be important in the pathogenesis of PCOS [56]. Later on, three studies specifically focused on the lipid metabolism have allowed to define a lipidomic profile in PCOS patients compared with control women at different stages of menstrual cycle [57], or suffering from obesity [58, 59].

In all these studies, however, the group of PCOS patients showed an important phenotypic heterogeneity, basically due to two factors: a) the variability in the diagnosis criteria, and b) the broad range of overlaid metabolic traits (dyslipemia, obesity, insulin resistance, cardiovascular disease). As pathophysiological mechanisms underlying the different PCOS phenotypes may differ from each other, these constraints can lead to differential metabolomic profiles not necessarily ascribed to the basic PCOS condition.

In this work, UPLC-QTOF-MS-based lipidomics approach was used to unravel changes in the plasma lipidome of PCOS patients. The aim of the study is to offer new insights in PCOS patients strictly selected in order to avoid confounding factors such as dyslipemia, obesity, altered glucose/insulin metabolism, cardiovascular disease, or cancer.

## RESULTS

### Clinical and metabolic characteristics

The clinical and metabolic characteristics of control and PCOS women are presented in Table 1.

**Table 1:** Anthropometric and metabolic parameters in healthy control subjects and PCOS patients

	Controls (*n* = 20)	PCOS (*n* =14)	*P* value
**Age (years)**	23.85 ± 5.63	24.14 ± 5.01	0.877
**BMI (kg/m2)**	20.78 ± 1.59	21.78 ± 2.77	0.362
**Waist (cm)**	76.50 ± 6.26	80.50 ± 9.41	0.127
**Systolic BP (mmHg)**	110.74 ± 14.46	112.93 ± 12.66	0.654
**Diastolic BP (mmHg)**	69.00 ± 10.81	71.50 ± 9.25	0.812
**Total cholesterol (mg/dl)**	163.00 ± 21.64	150.00 ± 33.22	0.494
**LDLc (mg/dl)**	85.00 ± 18.01	86.50 ± 24.59	0.528
**HDLc (mg/dl)**	61.00 ± 12.12	49.00 ± 9.52	0.051
**Triglycerides (mg/dl)**	63.15 ± 25.50	55.71 ± 21.40	0.379
**Glucose (mg/dl)**	82.50 ± 5.79	83.00 ± 8.79	0.806
**Insulin (mg/dl)**	5.65 ± 2.18	6.25 ± 1.99	0.969
**HOMA-IR (mg/dl)**	1.13 ± 0.45	1.25 ± 0.44	0.984
**HbA1c (%)**	5.08 ± 0.27	5.13 ± 0.25	0.783
**FSH (mIU/ml)**	3.90 ± 2.19	4.80 ± 1.44	0.054
**LH (mIU/ml)**	4.99 ± 4.90	5.72 ± 3.44	0.664
**Total testosterone (ng/ml)**	0.33 ± 0.12	0.53 ± 0.20	**0.002**
**Androstendione (ng/ml)**	3.35 ± 1.36	3.49 ± 1.37	0.506
**SHBG (nmol/l)**	81.05 ± 73.66	68.45 ± 31.83	0.455
**hsCRP (mg/l)**	1.12 ± 1.21	2.20 ± 2.34	0.103

Data are expressed as mean ± SD for parametric data and median (interquartile range) for non-parametric variables. Statistical significance (*p* < 0.05) was considered when compared by an unpaired Student's *t*-test or Mann-Whitney *U*-test for parametric or non-parametric data, respectively. Adjustment of variables by BMI was carried out using a univariate general linear model.

No statistically significant differences were found for age, BMI, waist, or blood pressure between PCOS and control women. Regarding the metabolic parameters, PCOS patients showed no changes in general lipid metabolism (total cholesterol, cholesterol-LDL, cholesterol-LDL, or triacylglycerides), glucose metabolism (glucose, HBA1c, insulin, or HOMA), endocrine system (FSH, LH, androstendione, or SHBG), and inflammatory marker (hsCRP) with respect to control subjects. Higher total testosterone levels (*p* < 0.002) in PCOS women than in controls was the only biochemical parameters significantly different.

### Plasma lipidomic signature of PCOS patients

The first goal of this study was to analyze global lipidomic differences between PCOS patients and healthy controls. Thus, we applied a non-targeted lipidomics approach focusing on the profiles of low molecular weight (m/z between 300 and 3000) ionizable lipid molecules. First, we detected 13380 features but after applied the MFE algorithm, 1320 features remained. Then, we filtered by frequency keeping those features present in at least 50% of the samples of each group, so 339 lipid species integrate the studied lipidome.

First of all, we analyzed whether specific lipid species correlate with the most used plasma biomarker of PCOS pathogenesis, total plasma testosterone, and also the only clinical parameter studied which revealed statistically different levels between groups (Table 1). The results indicate that 72 compounds statistically correlated with testosterone levels (Supplementary Table 2), suggesting a relationship between androgenic endocrine regulation and these lipid species.

**Figure 1:**
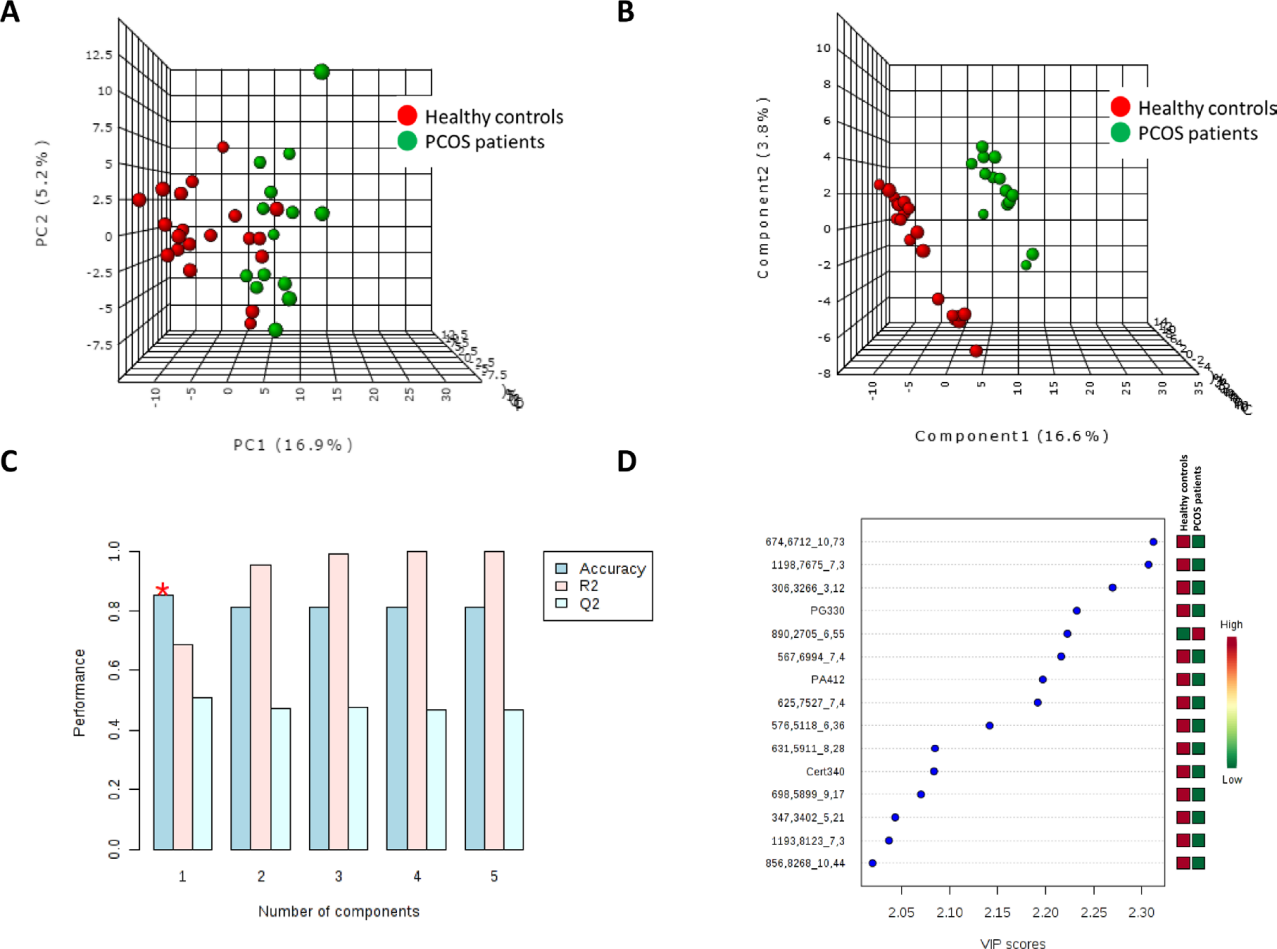
Multivariate statistics revealed the existence of a specific lipidomic signature of PCOS patients Both unsupervised Principal Component Analyses (PCA) (**A**) and supervised Partial Least Discriminant Analyses (s-PLS-DA) (**B**) indicate that it is possible to discriminate between healthy controls and PCOS patients basing on their plasma lipidome. (**C**) Cross validation (CV) analyses (10-fold CV method) indicates that we obtained the maximum accuracy using only one component. (**D**) Among the metabolites which most contribute to define the first component of PLS-DA we could identify (basing on exact mass, retention time, isotopic distribution and/or MSMS spectrum) the phosphatidylglyceride 33:0 (PG330), the phosphatidic acid 41:2 (PA412) and the ceramide t34:0 (Cert34:0). Unknown identities are represented as exact mass_retention time.

To determine whether the metabolite fingerprints in plasma differed between PCOS patients and healthy control subjects multivariate statistics were applied (Figure 1). Non-supervised PCA (Figure 1A) shows that there is an almost perfect clusterization of both groups suggesting a specific plasma lipidomic signature for PCOS subject without associated pathologies comparing to healthy control individuals. These results were confirmed by a supervised method such as PLS-DA (Figure 1B) where the both groups are perfectly discriminated. Cross validation values of PLS-DA model (Figure 1C) confirm that it is a good model to discriminate between PCOS and controls obtaining an accuracy value up to 0.8 and a maximum value of R^2^ using 3 components. The lower values of Q^2^ could be explained by the reduced number of individuals used in this study. After building the PLS-DA model, variable importance in projection (VIP) score was applied to rank the distinctive features based on their significance in discriminating between groups. Lipid molecular species with VIP score > 2 were selected as significant variables. Figure 1D shows the key differentiating lipid species sorted by increasing VIP score. Taking a VIP cut-off at 2, 15 molecular species were found to be significant discriminators between groups. Among them, we could identify (basing on exact mass, retention time, isotopic distribution and/or MS/MS spectrum) two glycerophospholipids (the phosphatidylglyceride 33:0, PG (33:0); and the phosphatidic acid 41:2, PA(41:2)) and a sphingolipid, the ceramide t34:0 (Cert34:0), all of them decreased in PCOS patients.

**Figure 2:**
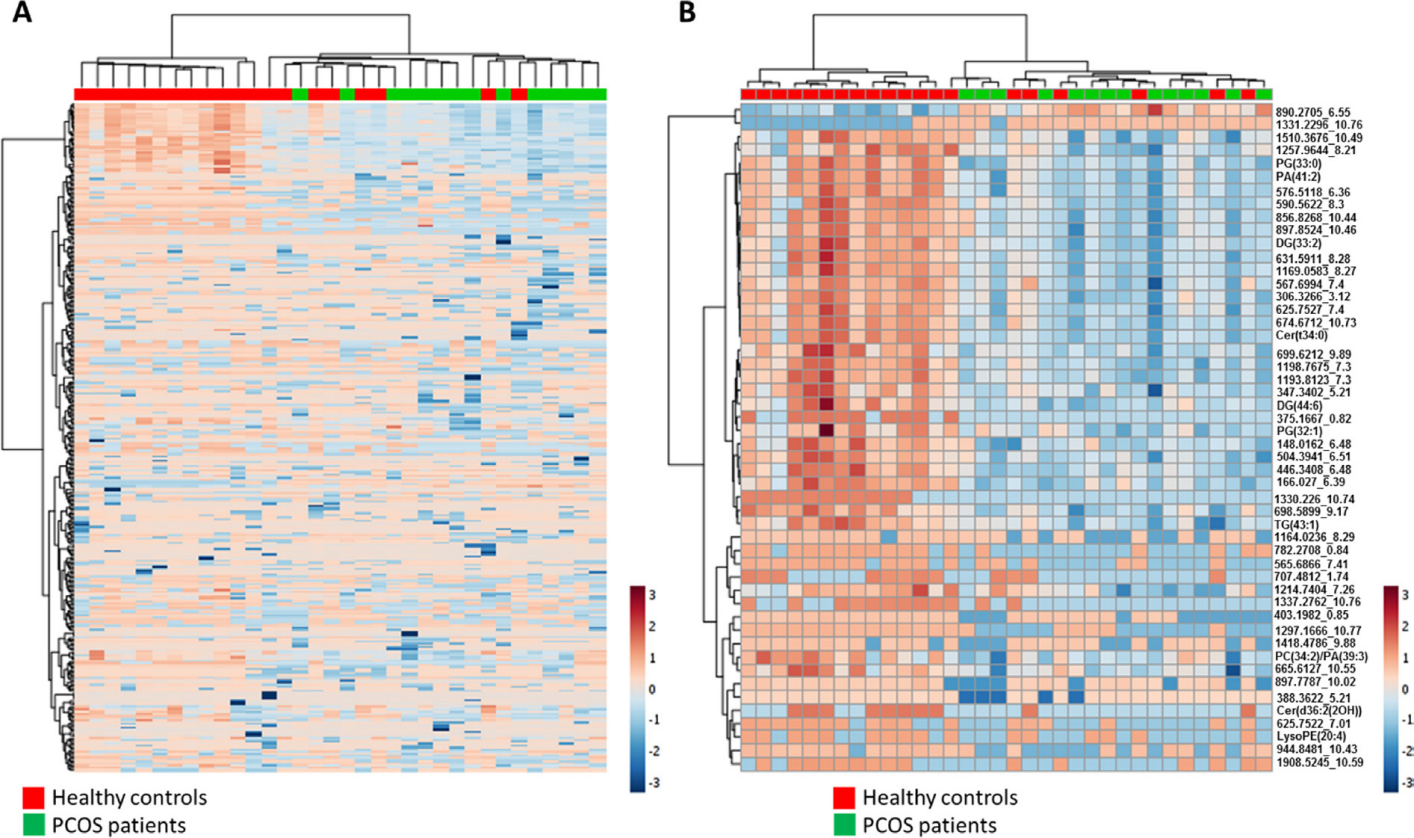
(**A**) Heat map representation of hierarchical clustering of 339 molecular features found in PCOS patients and healthy control plasma samples. (**B**) Heat map representations of hierarchical clustering analyses using 50 most statistical significant lipids species (*T*-test Unpaired with Benjamini Hochberg Correction) between PCOS and control samples. Unknown identities are represented as exact mass_retention time. Each line of this graphic represents an accurate mass ordered by retention time, coloured by its abundance intensity normalized to internal standard and baselining to median/mean across the samples. The scale from -7 (blue) to 7 (red) represents this normalized abundance in arbitrary units.

To better characterized plasma lipidomic signature of PCOS patients hierarchical clustering analyses using all 339 lipid species detected was performed (Figure 2A). This analysis reinforced the idea of a specific lipidomic signature of PCOS patients although a perfect separation is not reached.

After global characterization using multivariate statistics we wanted to describe changes in specific lipid species. To reach this goal, parametric *T* Test for unequal variances (*p* < 0.05, False Discovery Rate correction) was applied. Among 339 lipid species detected, 56 were statistically different between groups. 11 lipid species were identified (Table 2) and the remaining are still unidentified (Table 3).

**Table 2:** Identified compounds statistically different between groups (*T*-test unpaired with Benjamini Hochberg correction false discovery rate)

Lipid Family	Compounds	*p*.value	FDR	PCOS vs Healthy Controls	m/z	Retention Time	Product ion
**Glycero-phospholipids**	LysoPE(20:4)	4.49E-04	4.83E-03	down	502.2839	0.86	M+H+
	PA(39:3)	1.81E-04	2.27E-03	down	758.5633	7.63	M+NH4+
	PA(41:2)	7.51E-07	5.09E-05	down	771.584	6.36	M+H+
	PG(32:1)	1.03E-03	9.65E-03	down	721.5012	7.98	M+H+
	PG(33:0)	3.71E-07	4.20E-05	down	754.5572	6.36	M+NH4+
	PS(O-29:0)	5.17E-03	3.31E-02	down	680.4733	7.98	M+H+
**Sphingolipids**	Cer(t34:0)	2.98E-06	9.57E-05	down	556.5276	8.29	M+H+
**Glycerolipids**	DG(44:6)	6.82E-05	1.16E-03	down	763.5499	8.96	M+K+
	DG(33:2)	7.49E-05	1.21E-03	down	596.5205	8.3	M+NH4+
	DG(32:3)	3.38E-04	3.82E-03	down	580.5062	5.87	M+NH4+
	TG(43:1)	4.55E-04	4.83E-03	down	757.6426	9.18	M+Na+

Lipid species were identified by exact mass, retention time, isotopic distribution and MS/MS spectrum.

**Table 3:** Unidentified compounds statistically different between groups (*T*-test unpaired with Benjamini Hochberg correction false discovery rate)

Compounds	*p*.value	FDR	Mass	Retention Time	PCOS vs Healthy Controls
674.6712_10.73	7.87E-08	1.44E-05	674.6712	10.74	down
1198.7675_7.3	8.52E-08	1.44E-05	1198.7675	7.31	down
890.2705_6.55	5.45E-07	4.62E-05	890.2705	6.56	up
306.3266_3.12	9.90E-07	5.60E-05	306.3266	3.12	down
625.7527_7.4	1.24E-06	6.00E-05	625.7527	7.41	down
576.5118_6.36	1.71E-06	7.27E-05	576.5118	6.36	down
631.5911_8.28	3.10E-06	9.57E-05	631.5911	8.29	down
567.6994_7.4	2.78E-06	9.57E-05	567.6994	7.41	down
698.5899_9.17	5.76E-06	1.50E-04	698.5899	9.17	down
1193.8123_7.3	5.75E-06	1.50E-04	1193.8123	7.31	down
1169.0583_8.27	1.35E-05	3.28E-04	1169.0583	8.27	down
856.8268_10.44	1.79E-05	3.90E-04	856.8268	10.44	down
347.3402_5.21	1.84E-05	3.90E-04	347.3402	5.21	down
699.6212_9.89	2.65E-05	5.28E-04	699.6212	9.9	down
897.8524_10.46	3.89E-05	7.32E-04	897.8524	10.46	down
504.3941_6.51	4.89E-05	8.73E-04	504.3941	6.51	down
625.7522_7.01	7.95E-05	1.22E-03	625.7522	7.02	down
590.5622_8.3	8.54E-05	1.26E-03	590.5622	8.3	down
782.2708_0.84	1.18E-04	1.57E-03	782.2708	0.85	down
446.3408_6.48	1.20E-04	1.57E-03	446.3408	6.48	down
1330.226_10.74	1.19E-04	1.57E-03	1330.226	10.75	down
166.027_6.39	2.19E-04	2.65E-03	166.027	6.39	down
375.1667_0.82	3.38E-04	3.82E-03	375.1667	0.82	down
1257.9644_8.21	6.29E-04	6.46E-03	1257.9644	8.21	down
665.6127_10.55	8.87E-04	8.84E-03	665.6127	10.56	down
565.6866_7.41	9.76E-04	9.45E-03	565.6866	7.42	down
1908.5245_10.59	1.26E-03	1.09E-02	1908.5245	10.59	down
1510.3676_10.49	1.23E-03	1.09E-02	1510.3676	10.5	down
1337.2762_10.76	1.20E-03	1.09E-02	1337.2762	10.77	down
707.4812_1.74	1.41E-03	1.20E-02	707.4812	1.75	down
148.0162_6.48	1.56E-03	1.27E-02	148.0162	6.48	down
1331.2296_10.76	1.58E-03	1.27E-02	1331.2296	10.76	up
1214.7404_7.26	1.84E-03	1.45E-02	1214.7404	7.27	down
1297.1666_10.77	2.46E-03	1.90E-02	1297.1666	10.77	down
849.2429_6.54	3.31E-03	2.49E-02	849.2429	6.55	up
795.539_7.54	3.62E-03	2.61E-02	795.539	7.55	down
1566.5088_10.07	3.55E-03	2.61E-02	1566.5088	10.07	down
1612.4489_10.36	4.08E-03	2.82E-02	1612.4489	10.36	down
1418.4786_9.88	4.06E-03	2.82E-02	1418.4786	9.88	down
944.8481_10.43	4.20E-03	2.85E-02	944.8481	10.43	down
1164.0236_8.29	4.51E-03	3.00E-02	1164.0236	8.29	down
403.1982_0.85	5.05E-03	3.29E-02	403.1982	0.85	down
897.7787_10.02	6.20E-03	3.89E-02	897.7787	10.02	down
872.2602_8.96	7.76E-03	4.78E-02	872.2602	8.96	down
1328.2119_10.68	7.96E-03	4.82E-02	1328.2119	10.68	up

Unknown identities are represented as exact mass_retention time.

Among the identified lipids we found 6 lipids belonging to glycerophospholipids family, two belonging to sphingolipids and three glycerolipids. When a hierarchical analysis to statistically significant molecules was applied, we did not obtain a better clusterization than using the whole lipidome (Figure 2B).

**Figure 3:**
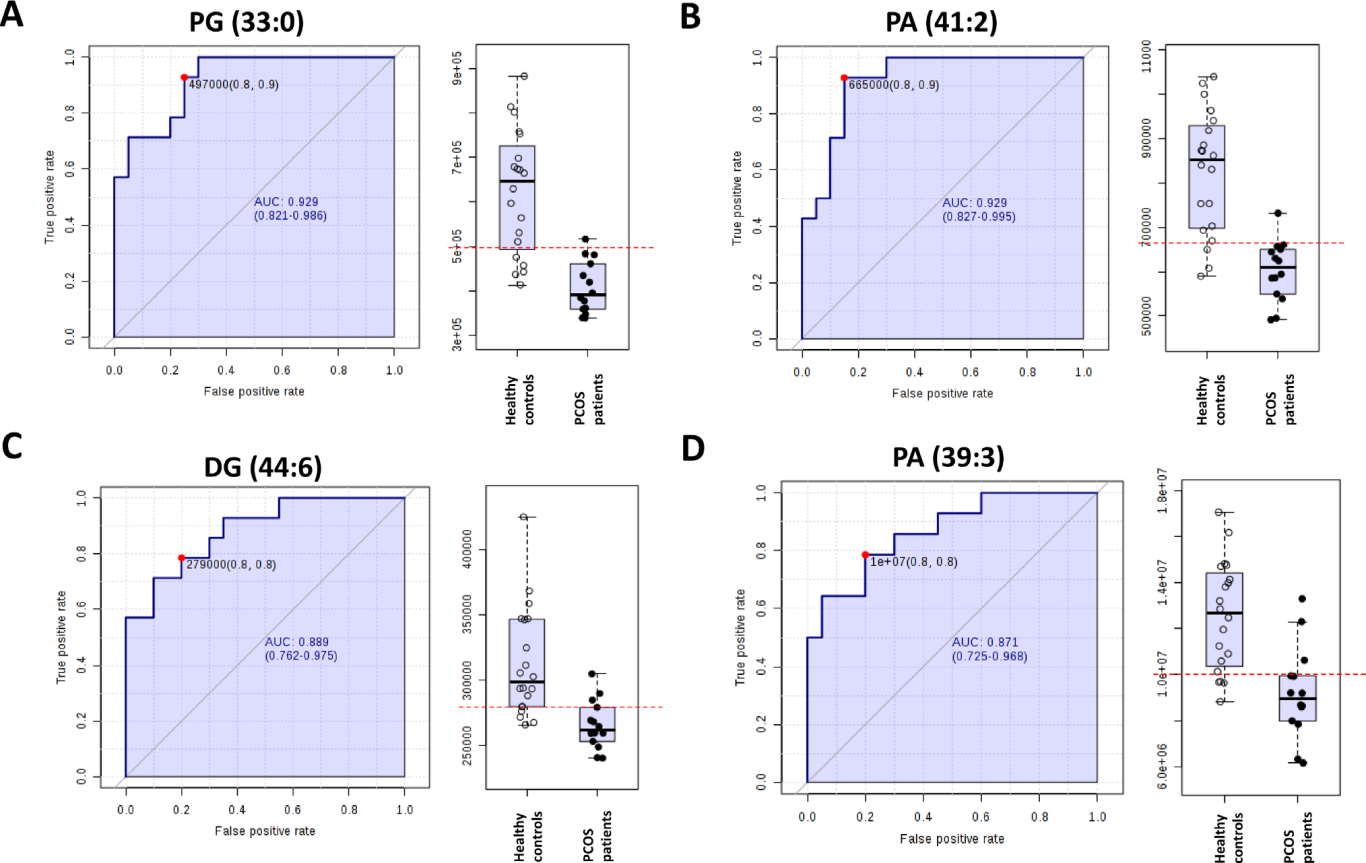
Receiver operating characteristic curve of (**A**) phosphatidylglicerol 33:0 (PG (33:0), (**B**) phosphatidic acid 41:2 (PA (41:2)), (**C**) diacylglycerol 44:6 (DG (44:6)) and (**D**) PA (39:3).

To further characterize the predictive value of these metabolites to discriminate PCOS condition, we performed ROC analyses using MS peak areas. Supplementary Table 3 shows the lipid species with an area under the curve (AUC) higher to 0.8. Among them, the identified molecules with a higher AUC (PG (33:0), PA (41:2), DG (44:6) and PA (39:3)) had lower levels in PCOS individuals than in healthy controls (Figure 3).

In addition with these changes, we also verified the existence of minor but significant differences in total plasma fatty acid profile between groups (Table 4). For particular fatty acids, there was a significant increase in the content of 14:0, and 24:0 in PCOS women; whereas 18:4n-3, 20:0, 20:1n-9, 20:2n-6, 20:5n-3, 22:4n-6, 22:5n-3, 24:1n-9, and 26:0 were decreased in PCOS group. These minor changes lead to a slight but significant change in the average chain length (ACL). Any additional change is verified for the other fatty acid indexes such as SFA, UFA, MUFA, PUFA, PUFAn-3, PUFAn-6, DBI, PI, or AI.

**Table 4:** Total fatty acid composition of plasma from control subjects and PCOS patients

	Control			PCOS			*P* value
**C14:0**	0.401	±	0.023	0.608	±	0.054	**0.001**
**C16:0**	20.563	±	0.469	21.553	±	0.370	0.130
**C16:1n-7**	1.149	±	0.093	1.351	±	0.130	0.203
**C18:0**	7.967	±	0.496	8.319	±	0.128	0.559
**C18:1n-9**	20.615	±	0.391	20.336	±	0.628	0.694
**C18:1n-7**	1.797	±	0.052	1.746	±	0.046	0.514
**C18:2n-6**	32.502	±	0.774	32.123	±	1.033	0.767
**C18:3n-3**	0.309	±	0.017	0.314	±	0.036	0.881
**C18:4n-3**	0.205	±	0.007	0.140	±	0.006	**0.001**
**C20:0**	0.302	±	0.013	0.160	±	0.006	**0.001**
**C20:1n-9**	0.276	±	0.013	0.232	±	0.009	**0.019**
**C20:2n-6**	0.067	±	0.009	0.041	±	0.003	**0.031**
**C20:3n-6**	1.502	±	0.080	1.405	±	0.086	0.425
**C20:4n-6**	6.626	±	0.300	6.534	±	0.365	0.847
**C20:5n-3**	0.055	±	0.004	0.043	±	0.003	**0.034**
**C22:0**	0.278	±	0.011	0.271	±	0.008	0.680
**C22:1n-9**	2.355	±	0.259	2.214	±	0.213	0.694
**C22:4n-6**	0.238	±	0.009	0.180	±	0.008	**0.001**
**C22:5n-6**	0.118	±	0.008	0.156	±	0.026	0.127
**C22:5n-3**	0.377	±	0.033	0.282	±	0.022	**0.038**
**C24:0**	0.055	±	0.004	0.128	±	0.017	**0.001**
**C22:6n-3**	1.615	±	0.085	1.518	±	0.115	0.493
**C24:1n-9**	0.387	±	0.017	0.283	±	0.011	**0.001**
**C26:0**	0.244	±	0.014	0.043	±	0.006	**0.001**
**ACL**	17.972	±	0.021	17.899	±	0.018	**0.019**
**SFA**	29.808	±	0.492	31.093	±	0.416	0.069
**UFA**	70.192	±	0.492	68.906	±	0.416	0.069
**MUFA**	26.577	±	0.507	26.163	±	0.654	0.616
**PUFA**	43.615	±	0.657	42.742	±	0.789	0.401
**PUFAn3**	2.562	±	0.100	2.303	±	0.156	0.154
**PUFAn6**	41.053	±	0.706	40.439	±	0.851	0.582
**DBI**	137.867	±	1.202	134.605	±	1.151	0.068
**PI**	81.355	±	1.275	78.730	±	1.371	0.178
**AI**	49.801	±	3.379	46.676	±	2.755	0.504

Abbreviations: ACL, average chain length; SFA, saturated fatty acids; UFA, unsaturated fatty acids; MUFA, monounsaturated fatty acids; PUFA, polyunsaturated fatty acids; PUFAn-3, polyunsaturated fatty acids series n-3; PUFAn-6, polyunsaturated fatty acids series n-6; DBI, double bond index; PI, peroxidizability index; AI, anti-inflammatory index.

## DISCUSSION

Previous metabolomic studies using plasma, serum, urine and follicular fluids suggested marked metabolic shifts associated with PCOS disease. In these studies, metabolomics techniques have been applied in PCOS patients suffering from obesity, insulin resistance, cardiovascular disease or treated with polytherapy in order to refine their molecular traits [44, 45, 47, 49–55]. The results suggested some particular changes in lipid profile, and different levels of free fatty acids, phosphoglycerides, and sphingolipids are recurrent findings. It is important to be in mind that human diseases such as obesity, diabetes and cardiovascular disease are linked with disturbances in lipid metabolism and, consequently, it could be very difficult to discern whether PCOS per se also shows alterations in lipid metabolism when PCOS patients with associated pathologies are studied.

Further, previous studies focused in PCOS patients strictly diagnosed by the basic triad (polycystic ovaries, amenorrhea and hyperandrogenism) in absence of associated pathologies suggested minor changes in lipid metabolism. Only slightly increased LDL-cholesterol and decreased HDL-cholesterol, as well as total cholesterol, have been described [57–59]. In this line, in our study (only focused in PCOS patients without associated pathologies) we did not find changes in the levels of triglycerides, total cholesterol, LDLc and HDLc, glucose levels, hemoglobin A1c, insulin, and HOMA index. However, this apparent low impact of PCOS pathology in lipid metabolism could be masked by limitations in the biochemical analysis where very few lipid species are analyzed (basically, triacylglycerides and cholesterol). In contrast, mass spectrometry (MS) and nuclear magnetic resonance (NMR)-based techniques have become powerful tools for high-throughput screening for lipid characterization giving us the possibility to describe the complete lipid profile (lipidome) within an organism, organ, tissue, cell, organelle, subcellular membrane or microdomain, and biological fluids. In this scenario, the human plasma lipidome is composed of more than 3000 confirmed lipid compounds which represent around 80% of the total human plasma metabolome [60].

In the present study we applied an LC-MS-based technique in order to describe specific changes in plasma lipidome which could be only attributed to PCOS physiopathology. The results demonstrated for the first time the existence of a plasma lipidomic profiling specifically associated with PCOS patients. This signature could be defined by 15 molecules including two glycerophospholipids (PG (33:0), PA (41:2)) and a sphingolipid (Cer(t34:0). Unfortunately, the other species are still unidentified.

Further, we found 56 lipid molecular species with different concentration (4 lipid species were up-regulated (7.1%), whereas 52 were down-regulated (92.9%)) in plasma from PCOS patients compared to healthy controls. Among them, 11 (19.3%) were identified (based on exact mass, retention time, isotopic distribution, and/or MS/MS spectrum) and different functions can be attributed.

The identified lipid species (11) belong to diverse lipid classes such as glycerolipids (5), glycerophospholipids (5), and sphingolipids (1), all of them down-regulated. One of the main components of cellular membranes are glycerophospholipids (GP), which are synthesized from glycerol-3-phosphate in a *de novo* pathway that primarily produces phosphatidic acid (PA) and diacylglycerol (DAG) or cytidine diphosphate-DAG (CDP-DAG). Via the *de novo* pathway, different types of GPs such as phosphatidylcholine (PC), phosphatidylethanolamine (PE), phosphatidylserine (PS), phosphatidylinositol (PI), phosphatidylglycerol (PG), and cardiolipin (CL) are generated. Subsequently, GP acyl chains are remodeled, via generation of lysoglycerophospholipids, by regulated and coordinated reactions of diverse enzymes [61]. Thus, the decreased content observed in PCOS patients of different lipid species such as PA (2), DAG (3), and even TAG (1), PG (2), PS(1), and LPE(1) suggest that PCOS is associated with a lower *de novo* biosynthesis pathway of glycerophospholipids and lower remodeling activity of PE likely due to the hyperandrogenism ascribed to PCOS. In addition, the decreased content in the identified ceramide (1) is probably also linked to a decreased *de novo* synthesis induced by PCOS status instead of an increase in degradation pathway. Globally, the down-regulation of the identified lipid species likely due to a decreased activity of the biosynthesis pathway along with the minor changes in the total plasma fatty acid composition could explain the not alterations o minor changes for LDL and HDL levels described in PCOS patients. This particular lipid profile can also have additional effects derived from their role as lipid second messengers for some of them such as DAGs and ceramides. These molecules have been linked to obesity, insulin resistance, and metabolic disorders [62]. Consequently, the decrease in some of the DAGs may be a reason why these PCOS patients have not developed any of the metabolic diseases associated yet. According to our results, this pathway could be differentially regulated in PCOS patients and affected at long-term during the natural history of the disease and be involved in infertility and pregnancy loss, two main clinical consequences of this pathology [63, 64]. An interesting additional observation derived from our study is that free fatty acids (FFAs) are not differential lipid species between PCOS women and healthy control, in contrast to several previous metabolomics and lipidomics studies where systematic increases in free fatty acid contents are described [44, 45, 50, 51, 53, 54, 65]. FFAs are important for physiological homeostasis, providing a major portion of mammalian bioenergetics needs. In addition, FFAs are the substrate by which TAG stored in adipose tissue is transported to its sites of consumption, being the adipose tissue the only significant site of FFA release into plasma [66]. Therefore, the results of our study demonstrate that the described changes in lipid species can be ascribed specifically to the PCOS condition, whereas the described changes in FFAs in most studies are derived from the obesity associated to PCOS women.

In conclusion, our study demonstrates that PCOS per se is a pathological entity that also presents alterations, though modest, in lipid metabolism. Although sample size is small, and should be validated in other cohorts, the accuracy in the elected group ensures and offers valuable information about the physiopathology of PCOS. Furthermore, our study confirms that using a lipidomic approach it is able to discriminate a specific phenotype from PCOS women without others associated pathologies.

## MATERIALS AND METHODS

### Subjects

The study was conducted in the Service of Endocrinology at the University Hospital Dr. Peset (Valencia, Spain). Fourteen women with PCOS and 20 controls were selected according to age and body mass index (BMI). Table 1 shows anthropometric and metabolic parameters in control subjects and PCOS patients participating in this study. Controls were volunteers recruited from the University Hospital Dr Peset and the Faculty of Medicine (Valencia University, Valencia, Spain). PCOS subjects were diagnosed using the Rotterdam criteria [13], which are the following: oligoovulation (cycles longer than 35 days or less than 26 days) [5]; elevated free testosterone levels (>0.5 ng/dl; the cut-off level for free testosterone was the mean ± 2 SD according to normal levels in controls); hirsutism (total Ferriman-Gallwey score > 7) and polycystic ovaries (presence of 12 or more small -2 to 9 mm- follicles in each ovary), identified by trans-vaginal ultrasonography. Ultrasound scans were performed and scored independently by one of two experienced and blinded reviewers. None of the subjects had any systemic or endocrine disease or galactorrhea, or any condition affecting her reproductive physiology. Exclusion criteria were active infectious diseases, malignant neoplasia, diabetes mellitus, anemia, thromboembolism, history of ischaemic heart disease, stroke and the taking of lipid-lowering or antihypertensive drugs. Absence during the previous semester of any medication that might have affected the hypothalamic-pituitary-gonadal axis was confirmed in all subjects. The study was approved by the ethics committee of the University Hospital Dr. Peset and was performed in accordance with the Helsinki declaration. Informed consent of all participants was obtained as required by these institutions.

### Biochemical determinations

An anthropometric evaluation was performed in all subjects in whom weight (kg), height (m) and waist circumference (cm) were measured. Body mass index was then calculated (BMI = weight (kg)/height (m)^2^). After 12 hours of fasting, blood was collected from the antecubital vein at 8–10 a.m, on the second/third day of the menstrual cycle (follicular phase) or after 3 months of amenorrhea. In subjects with very irregular cycles, blood was collected after progesterone deprivation. Samples were processed immediately in order to avoid haemolysis and were frozen until analysis.

Total cholesterol and triglycerides were measured using enzymatic assays. Concentration of low density lipoproteins cholesterol (LDLc) was calculated using the Friedewald formula, and high density lipoproteins cholesterol (HDLc) levels were recorded using a direct method with a Beckman LX-20 autoanalyser (Beckman Coulter, La Brea, CA, USA). The intraserial variation coefficient was <3.5 % for all determinations. An Automatic Glycohemoglobin Analyzer was employed to assess HbA1c (Arkray, Inc., 73 KYOTO, Japan). Glucose levels were measured with a Dax-72 autoanalyzer using enzymatic techniques (Bayer Diagnostic, Tarrytown, NY, USA). Insulin concentration was determined by means of an enzymatic luminescence technique. High sensitivity C-reactive protein (hsCRP) levels were assessed by an immunonephelometric assay (Behring Nephelometer II, Dade Behring, Inc., Newark, DE, USA) with an intra-assay coefficient of variation of 8.7% and sensitivity of 0.01 mg/L. IR was calculated by homeostasis model assessment (HOMA) using baseline glucose and insulin: HOMA = (fasting insulin (μU/ml) × fasting glucose (mmol/L)/22.5. Serum luteinizing hormone (LH) and follicle-stimulating hormone (FSH) were measured using a 2-site monoclonal non-isotopic system (Architect, Abbott Laboratories, Abbott Park, IL). Sex hormone binding globulin (SHBG), androstendione and testosterone were measured in our hospital's Clinical Analysis Service using specialized chemiluminiscence techniques.

### Lipidomic analysis

Chemicals. Synthetic lipids were obtained from Avanti Polar Lipids Inc. (Alabaster, AL, USA) and Sigma-Aldrich (Madrid, Spain). Fatty acid methyl ester standards were obtained from Larodan Fine Chemicals (Mälmo, Sweden) and from Sigma-Aldrich (Madrid, Spain). Methyl tert-butyl ether (MTBE) LC-MS, acetonitrile LC-MS, isopropanol LC-MS, potassium chloride, chloroform, ammonium formate and ammonium hydroxide were purchased from Sigma-Aldrich (Madrid, Spain); methanol was from Carlo Erba (Milano, Italy); acetone was from Riedel-de-Häen (Seelze, Germany); and LC/MS-grade isopropanol and formic acid were from Baker (Phillipsburg, NJ, USA).

### Lipidomic analysis: plasma lipidome

Preparation of Lipid Standards. Lipid standards consisting of isotopically labeled lipids (see Supplementary Table 1) were used for external standardization (i.e. lipid family assignment) and internal standardization (i.e. for adjustment of potential inter- and intra-assay variances). Stock solutions were prepared by dissolving lipid standards in MTBE at a concentration of 1 mg/mL and working solutions were diluted to 2.5 μg/mL in MTBE.

Lipid extraction. Lipidomic analysis was based on a previously validated method [67]. Briefly, in order to precipitate plasma protein fraction, 5 μl of miliQ water and 20 μl of methanol were added to 10 μl of plasma sample. After the addition, samples were vigorously shaken for 2 min. Then, for lipid extraction, 250 μl of MTBE (containing internal lipid standards) were added and samples were immersed in a water bath (ATU Ultrasonidos, Valencia, Spain) with an ultrasound frequency and power of 40 kHz and 100 W, respectively, at 10°C for 30 min. Then, 75 μL of miliQ water were added to the mixture and organic phase was separated by centrifugation (1,400 g) at 10°C for 10 min. Lipid extracts, contained in the upper phase, were collected and subjected to mass-spectrometry. A pool of all lipid extracts was prepared and used as quality controls (QC) as previously described [68].

LC-MS/MS method. Lipid extracts were subjected to liquid chromatography coupled to mass-spectrometry (LC-MS) using an Agilent UPLC 1290 coupled to the Q-TOF MS/MS 6520 (Agilent Technologies, Barcelona, Spain) basing on previously published method [69]. Sample compartment was refrigerated at 4°C and, for each sample, 10 μl of lipid extract was applied onto 1.8 μm particle 100 × 2.1 mm id Waters Acquity HSS T3 column (Waters, Mildord, MA, USA) heated at 55°C. The flow rate was 400 μl/min with solvent A composed of 10mM ammonium acetate in acetonitrile-water (40:60, v/v) and solvent B composed of 10 mM ammonium acetate in acetonitrile-isopropanol (10:90, v/v). The gradient started at 40% B and reached 100% B in 10min and held for 2 min. Finally, the system was switched back to 60% B and equilibrated for 3 min. Duplicate runs of the samples were performed to collect positive and negative electrospray ionized lipid species in a TOF mode, operated in full-scan mode at 100 to 3000 m/z in an extended dynamic range (2 GHz), using N2 as nebulizer gas (5 L/min, 350°C). The capillary voltage was set 3500 V with a scan rate of 1 scan/s. Continuous infusion using a double spray with masses 121.050873, 922.009798 (positive ion mode) and 119.036320, 966.000725 (negative ion mode) was used for in-run calibration of the mass spectrometer. For MS/MS analyses we applied a previously described method [70].

Data Analyses. The MassHunter Data Analysis Software (Agilent Technologies, Barcelona, Spain) was used to collect the results and the MassHunter Qualitative Analysis Software (Agilent Technologies, Barcelona, Spain) to obtain the molecular features of the samples, representing different, co-migrating ionic species of a given molecular entity (i.e. ion adducts) using the Molecular Feature Extractor algorithm (Agilent Technologies, Barcelona, Spain) [71]. This algorithm is a compound-finding technique that locates individual sample components (molecular features), even when chromatograms are complex and compounds are not well resolved. MFE locates ions that are covariant (rise and fall together in abundance), but the analysis is not exclusively based on chromatographic peak information. The algorithm uses the accuracy of the mass measurements to group related ions, related by charge-state envelope, isotopic distribution, and/or the presence of adducts and dimers. It assigns multiple species (ions) that are related to the same neutral molecule (for example, ions representing multiple charge states or adducts of the same neutral molecule) to a single compound that is referred to as a feature. Using this approach, the MFE algorithm can locate multiple compounds within a single chromatographic peak. We selected samples with a minimum absolute abundance of 5000 counts and with a minimum of 2 ions. Multiple charge states were not considered. Compounds from different samples were aligned using a RT window of 0.1% ± 0.15 min and a mass window of 5.0 ppm ± 2.0 mDa. Only common features (found in at least 50% of the samples of the same condition) were analyzed, correcting for individual bias and excluding possible contaminants and artefacts. Finally, the MassHunter Mass Profiler Professional Software (Agilent Technologies, Barcelona, Spain) was used to perform a non-targeted lipidomic analysis over the extracted features. Only common features (found in at least 50% of the samples of the same condition) were taken into account to correct for individual bias. Multivariate statistics (Hirerchical Clustering, PCA and PLS-DA analyses) were done using both MassHunter Mass Profiler Professional and Metaboanalyst softwares. Variable importance in projection (VIP) score was calculated using Metaboanalyst software [72, 73]. The masses representing significant differences by ANOVA (*p* < 0.05 with Benjamini-Hochberg Multiple Testing Correction) were searched against the LIPID MAPS database (The LIPID MAPS Lipidomics Gateway, http://www.lipidmaps.org/, May 2014) (exact mass ppm <20) and the MS/MS spectra were checked using the LipidBlast software [74].

### Lipidomic analysis: plasma fatty acids

Fatty acid preparation. After lipid extraction, fatty acyl groups were analyzed as methyl esters derivatives by gas chromatography (GC) [70]. Briefly, fatty acids were transesterified by incubation in 2 ml of 5% methanolic HCl at 75°C for 90 min. The resulting fatty acid methyl esters (FAMEs) were extracted by adding 2 ml of n-pentane and 1 ml of saturated NaCl solution. The n-pentane phase was separated, evaporated under N_2_ gas, re-dissolved in 80 μl of carbon disulfide and 2 μl were used for GC analysis.

GC method. The analysis was performed on a GC System 7890A with a Series Injector 7683B and a flame ionization detector (FID) (Agilent Technologies Inc., Barcelona, Spain) equipped with a DBWAX capillary column (length 30 m × inner diameter 0.25 mm × film thickness 0.20 μm; Agilent Technologies Inc., Barcelona, Spain). The injections were performed in the splitless mode. The temperature of the injector was 220°C. The flow rate of helium (99.99%) carrier gas was maintained at a constant rate of 1.8 ml/min. The column temperature was held at 145°C for 5 min; subsequently, the column temperature was increased by 2°C/min to 245°C for 50 min, and held at 245°C for 10 min, and with a post-run of 250°C for 10 min.

Data analysis. Identification of the twenty-four FAMEs was made by comparison with authentic standards. Results were expressed as mol%. The fatty acid profile detected, identified and quantified represents more than 95% of the total chromatogram. The following fatty acid indexes were calculated: saturated fatty acids (SFA); unsaturated fatty acids (UFA); monounsaturated fatty acids (MUFA); polyunsaturated fatty acids (PUFA) from n-3 and n-6 series (PUFAn-3 and PUFAn-6); average chain length (ACL) = [(Σ%Total14 × 14) + (Σ%Total16 × 16) + (Σ%Total18 × 18) + (Σ%Total20 × 20) + (Σ%Total22 × 22)+ (Σ%Total24 × 24)]/100]; double bond index (DBI) = [(1 × Σmol% monoenoic) + (2 × Σmol% dienoic) + (3 × Σmol% trienoic) + (4 × Σmol% tetraenoic) + (5 × Σmol% pentaenoic) + (6 × Σmol% hexaenoic)]; peroxidizability index (PI) = [(0.025 × Σmol% monoenoic) + (1 × Σmol% dienoic) + (2 × Σmol% trienoic) + (4 × Σmol% tetraenoic) + (6 × Σmol% pentaenoic) + (8 × Σmol% hexaenoic)]; and anti-inflammatory index (AI): [[(20:3n-6) + (20:5n-3) + (22:6n-3)]/(20:4n-6)]*100.

Statistics. Comparisons between groups were analyzed with one way ANOVA followed by DMS tests. These statistical analyses were performed using the SPSS software (SPSS, Chicago, IL, USA). The level of statistical significance was set at *p* < 0.05 in all the analyses.

## SUPPLEMENTARY MATERIALS TABLES







## Abbreviations

ACL: Average Chain Length
BMI: Body Mass Index
BP: Blood Pressure
CER: Ceramide
DAG: Diacylglyceride
DBI: Double Bond Index
FA: Fatty Acid
FSH: Follicle-Stimulating Hormone
HOMA-IR: Homeostatic Model Assessment-Insulin Resistance
HDL: High Density Lipoprotein
hsCRP: high sensitivity C-Reactive Protein
LC-ESI-QTOF MS/MS: Liquid Chromatography-Electrospray Ionization-Quadrupole Time of Flight Mass Spectrometry/Mass Spectrometry
LDL: Low Density Lipoprotein
LH: Luteinizing Hormone
MUFA: Monounsaturated Fatty Acid
PC: Phosphatidylcholine
PCA: Principal Component Analysis
PCOS: Polycystic Ovary Syndrome
PE: Phosphatidylethanolamine
PLS-DA: Partial Least Square-Discrimination
PI: Peroxidizability Index
PUFA: Polyunsaturated Fatty Acid
PS: Phosphatidylserine
SFA: Saturated Fatty Acid
SHBG: Sex Hormone-Binding Globulin
SP: Sphingolipid
TAG: Triacylglyceride
UFA: Unsaturated Fatty Acid.
