# Supplementary material for: Lipidomics reveals altered biosynthetic pathways of glycerophospholipids and cell signaling as biomarkers of the polycystic ovary syndrome
Source: Oncotarget. 2017 Dec 17;9(4):4522–36. doi: 10.18632/oncotarget.23393 (PMC5796992; doi:10.18632/oncotarget.23393)
Supplement: Supplementary file 1 [file oncotarget-09-4522-s001.pdf]

Lipidomics reveals altered biosynthetic pathways of glycerophospholipids and cell signaling as biomarkers of the polycystic ovary syndrome

SUPPLEMENTARY MATERIALS

Supplementary Table 1: Class representative and extraction internal standards added to the samples

Compound	Reference
1,3(d5)-dihexadecanoyl-glycerol	110537, Avanti Polar Lipids
1,3(d5)-dihexadecanoyl-2-octadecanoyl-glycerol	110543, Avanti Polar Lipids
1-hexadecanoyl(d31)-2-(9Z-octadecenoyl)-sn-glycero-3-phosphate	110920, Avanti Polar Lipids
1-hexadecanoyl(d31)-2-(9Z-octadecenoyl)-sn-glycero-3-phosphocholine	110918, Avanti Polar Lipids
1-hexadecanoyl(d31)-2-(9Z-octadecenoyl)-sn-glycero-3-phosphoethanolamine	110921, Avanti Polar Lipids
1-hexadecanoyl-2-(9Z-octadecenoyl)-sn-glycero-3-phospho-(1’-rac-glycerol-1′,1′,2′,3′,3′-d5)	110899, Avanti Polar Lipids
1-hexadecanoyl(d31)-2-(9Z-octadecenoyl)-sn-glycero-3-phospho-myo-inositol	110923, Avanti Polar Lipids
1-hexadecanoyl(d31)-2-(9Z-octadecenoyl)-sn-glycero-3-[phospho-L-serine]	110922, Avanti Polar Lipids
26:0-d4 Lyso PC	860389, Avanti Polar Lipids
18:1 Chol (D7) ester	111015, Avanti Polar Lipids
cholest-5-en-3ß-ol(d7)	LM-4100, Avanti Polar Lipids
D-erythro-sphingosine-d7	860657, Avanti Polar Lipids
D-erythro-sphingosine-d7-1-phosphate	860659, Avanti Polar Lipids
N-palmitoyl-d31-D-erythro-sphingosine	868516, Avanti Polar Lipids
N-palmitoyl-d31-D-erythro-sphingosylphosphorylcholine	868584, Avanti Polar Lipids
Octadecanoic acid-2,2-d2	19905-58-9, Sigma Aldrich

Supplementary Table 2: Pearson correlation analyses between specific lipid species and total testosterone plasma levels of subjects. See Supplementary_Table_2

Supplementary Table 3: Lipid species with an area under the curve higher than 0.8 in receiver operator characteristic (ROC). See Supplementary_Table_3
